# Role of lipid transporters in fungal physiology and pathogenicity

**DOI:** 10.1016/j.csbj.2019.09.001

**Published:** 2019-09-04

**Authors:** Juliana Rizzo, Lyubomir Dimitrov Stanchev, Vanessa K.A. da Silva, Leonardo Nimrichter, Thomas Günther Pomorski, Marcio L. Rodrigues

**Affiliations:** a Instituto de Microbiologia Paulo de Góes (IMPG), Universidade Federal do Rio de Janeiro (UFRJ), Rio de Janeiro, Brazil; b Department of Molecular Biochemistry, Ruhr University Bochum, Faculty of Chemistry and Biochemistry, 44780 Bochum, Germany; c Department of Plant Biology and Biotechnology, University of Copenhagen, Thorvaldsensvej 40, 1871 Frederiksberg C,Denmark; d Programa de Pós-Graduação em Biologia Parasitária do Instituto Oswaldo Cruz (IOC), Fundação Oswaldo Cruz (Fiocruz), Rio de Janeiro, Brazil; e Instituto Carlos Chagas, Fundação Oswaldo Cruz (Fiocruz), Curitiba, Brazil

**Keywords:** Flippases, ABC transporters, Fungal infections, Antifungals, Phosphatidylserine, Phosphatidylethanolamine, Sterols

## Abstract

The fungal cell wall and membrane are the most common targets of antifungal agents, but the potential of membrane lipid organization in regulating drug-target interactions has yet to be investigated. Energy-dependent lipid transporters have been recently associated with virulence and drug resistance in many pathogenic fungi. To illustrate this view, we discuss (i) the structural and biological aspects of ATP-driven lipid transporters, comprising P-type ATPases and ATP-binding cassette transporters, (ii) the role of these transporters in fungal physiology and virulence, and (iii) the potential of lipid transporters as targets for the development of novel antifungals. These recent observations indicate that the lipid-trafficking machinery in fungi is a promising target for studies on physiology, pathogenesis and drug development.

## Graphical abstract

**Figure:**
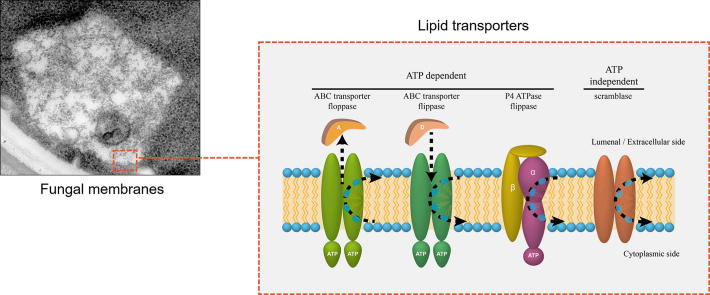

## 1 Introduction

Fungal infections kill more than 1.5 million people every year [1,2]. Despite the high mortality rates, diseases caused by fungi are still underappreciated by decision makers and the general public, representing, therefore, a major problem of public health [3]. There are only four major classes of antifungal drugs currently in clinical and agricultural use: azoles (inhibitors of ergosterol synthesis, a major plasma membrane component), polyenes (ergosterol-binding compounds), echinocandins (inhibitors of β-1,3-glucan synthesis), and *pyrimidine analogues* (inhibitors of nucleic acid synthesis). These drug classes are ineffective in a number of cases, which is linked to toxicity, low bioavailability in target tissues and antifungal resistance [4]. In this scenario, morbidity and mortality rates due to fungal infections remain high, which highlights the need for studies on new antifungal targets and compounds [3].

Fungal membranes and membrane-associated biosynthetic and metabolic pathways are important targets for antifungal compounds and prophylactic strategies [[5], [6], [7], [8], [9]]. Composed of a double layer of lipids, cellular membranes provide a permeability barrier and an interface between the interior and exterior of a cell and between compartments within the cell. Each membrane is composed of hundreds of different lipid species and has its own characteristic composition and dynamics [10]. For instance, lipids in the eukaryotic plasma membrane, the trans-Golgi network, endosomes and secretory vesicles are asymmetrically arranged between the two leaflets, with the aminophospholipids phosphatidylserine (PS) and phosphatidylethanolamine (PE) restricted to the cytosolic leaflet [11,12].This asymmetric lipid arrangement provides different characteristics to both sides of the membrane and plays a crucial role in multiple cellular processes, including regulation of membrane traffic, cell division, lipid metabolism, and lipid signaling [11,13,14].

**Fig. 1:**
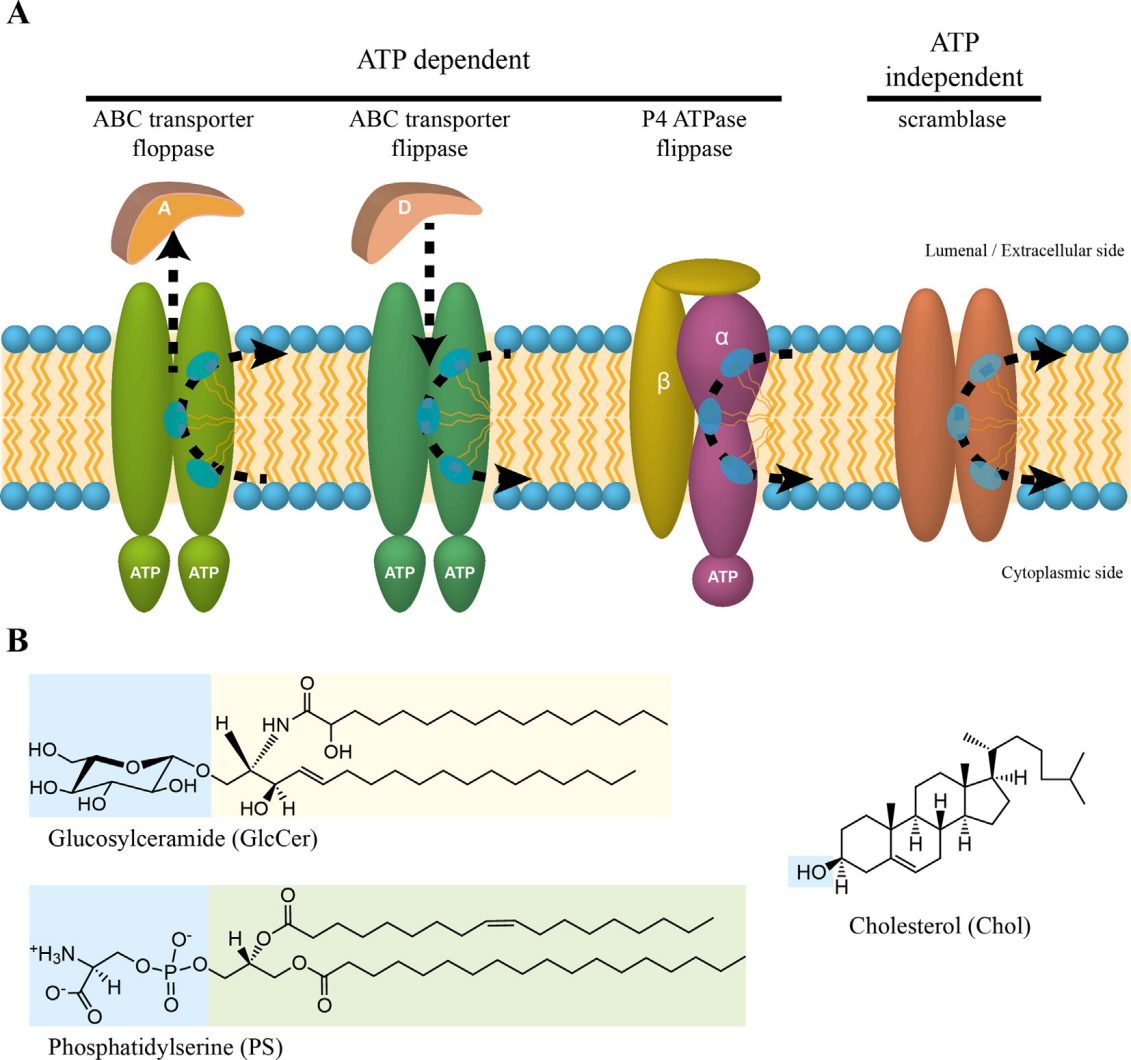
Lipid transporters and membrane lipid asymmetry. A) ATP-dependent transporters of the P4 ATPase and ABC transporter families can maintain an asymmetric phospholipid distribution by moving specific lipids towards (flippase) or away from the cytosolic leaflet (floppase). Some ABC transporters may also function in less obvious ways to translocate lipids by controlling their insertion into the plasma membrane upon their passage across the cell wall via donor binding proteins (D) and/or by facilitating their removal from the plasma membrane to extracellular acceptor proteins (A). Cellular activation triggered by cytosolic calcium, caspases or other stimuli can collapse the lipid asymmetry by the transient activity of ATP-independent scramblases, which can translocate lipids bidirectionally across the membrane. B) The structures of glucosylceramide, phosphatidylserine, and cholesterol, which are lipid transporter substrates and belong to the sphingolipids, glycerophospholipids, and sterols classes, respectively. The polar head groups are shaded blue, while the common backbone of glycerophospholipids and sphingolipids is shaded green and yellow, respectively. (For interpretation of the references to colour in this figure legend, the reader is referred to the web version of this article.)

Current data support a role of different groups of lipid transporters in establishing and regulating the asymmetric distribution of lipids between the two leaflets of cellular membranes. These transporters can be classified into two categories: (i) ATP-driven transporters that actively translocate specific lipids from one leaflet to the other, catalyzing inward or outward phospholipid movement across cellular membranes and (ii) ATP-independent transporters, also called scramblases, that facilitate a rapid bi-directional movement of lipids between the two plasma membrane leaflets, disrupting the lipid asymmetry created by the ATP-dependent transporters [15,16]. These two categories of lipid transporters and the chemical structures of some of their substrates are illustrated in Fig. 1 (A and B, respectively). Given their critical roles in fungal physiology, these transporters might be a promising therapeutic target for antifungal development. This review is focused on the key role played by ATP-driven lipid transporters in fungal physiology and pathogenicity. We will summarize recent information on this topic and provide an overview of their biological functions and of what is known about lipid transporters in pathogenic fungi.

## 2 Lipid transport catalyzed by P-type ATPases and ABC transporters

ATP-dependent lipid transporters in the eukaryotic plasma membrane are generally membrane proteins that belong to the family of P-type ATPases, or the family of ATP-binding cassette (ABC) transporters. These transporters use the energy of ATP hydrolysis to catalyze the transbilayer movement of a variety of lipids [16].

**Fig. 2:**
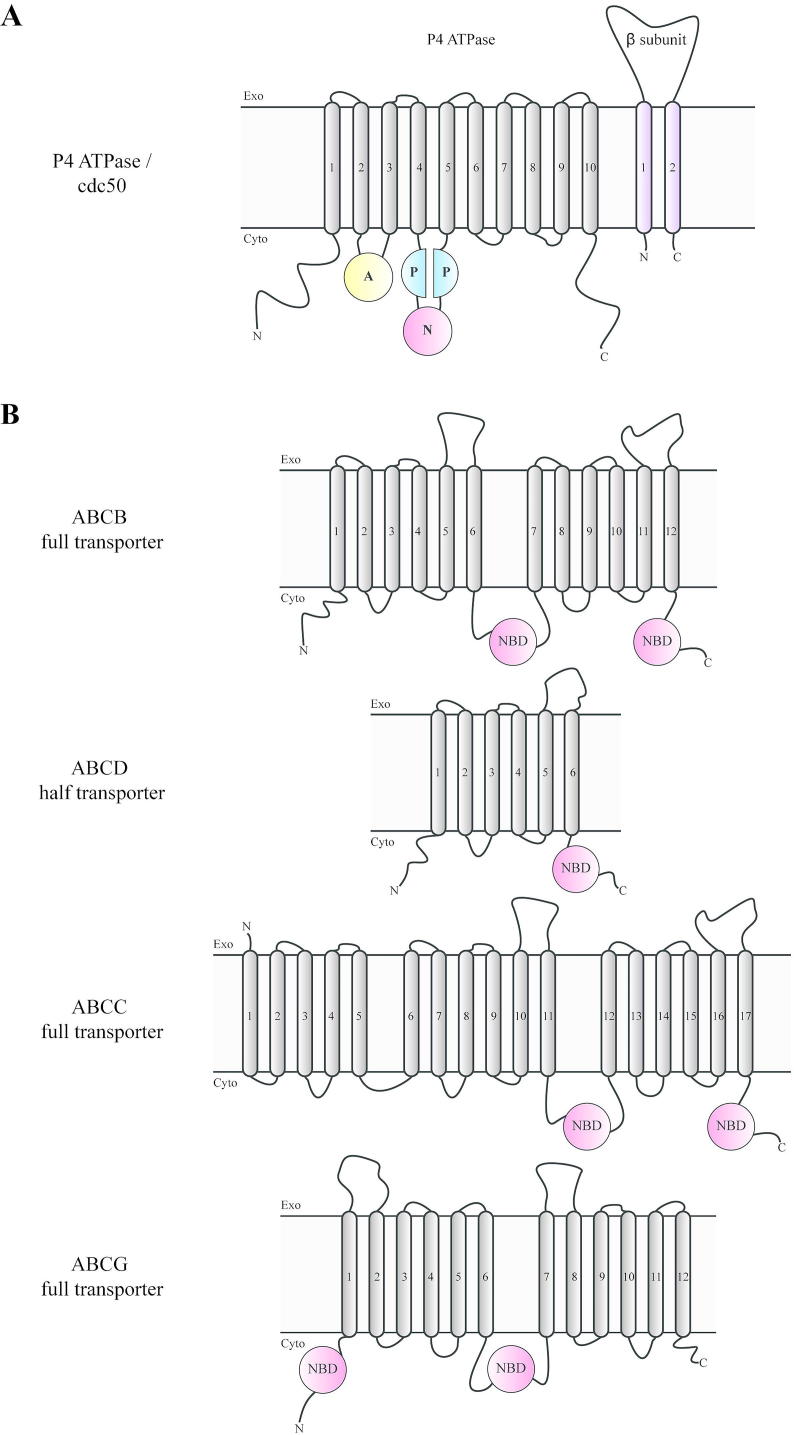
Topology of P4 ATPase and ABC transporters. A)P4 ATPases consist of one transmembrane domain with ten transmembrane helices labeled 1 to 10. The cytosolic domain of the transporter is divided into three major domains; the actuator domain (A), the nucleotide binding domain (N)and the phosphorylation domain (P)shown in yellow, blue, and red, respectively. Many P4 ATPases form a heteromeric complex with a β-subunit consisting of two transmembrane spans and a large exoplasmic loop. B)ABC transporters differ in the number of transmembrane domains (TMDs, indicated as numbered boxes) and nucleotide binding domains (NBDs, shown in red). They can occur as one complete transporter or two half-transporters. The functional unit always comprises two nucleotide-binding domains (NBD) present on the cytosolic side of the membrane. NBD1 is either situated at the C-terminal end of one-half transporter or is connecting TMD1 and TMD2 in the full transporter; alternatively, the domain architecture can have a reverse topology, i.e. NBD1-TMD1-NBD2-TMD2. (For interpretation of the references to colour in this figure legend, the reader is referred to the web version of this article.)

P-type ATPases constitute a large superfamily of active membrane pumps. Based on sequence similarity, the P-type ATPase family is divided into five subfamilies with different transport specificities, among which P4 ATPases are lipid flippases [17]. These enzymes translocate specific lipids in a stereoisomer specific manner from the luminal to the cytoplasmic side of cellular membranes [14,18]. All P4 ATPases have a similar structural organization consisting of a membrane domain typically comprised of 10 transmembrane segments, which serves as the pathway for translocation of lipid substrates across cell membranes **(**Fig. 2A**)**. Three cytoplasmic domains are involved in the ATP catalytic cycle: the nucleotide or N-domain binding ATP, the phosphorylation or P-domain and the actuator or A-domain with a conserved DGET motif that facilitates the dephosphorylation of the phosphorylated intermediate,hence the designation P type [17]. The cytosolic A- and P-domains are directly linked to transmembrane segments in the M-domain, whereas the N-domain is an insertion within the P-domain. The cytosolic amino and carboxy termini of P-type ATPases vary in length, and often contain additional regulatory domains or motifs.

P4 ATPases are unique to eukaryotes and are found in every eukaryotic genome that has been sequenced so far, whereas they are absent from eubacteria and archaea [18]. Most family members are known to associate with an accessory subunit known as Cdc50 proteins, resulting in a heterodimeric complex. The recently described cryo-electron microscopy-derived structures of the P4 ATPase in complex with its subunit revealed a tight association of both proteins, with the subunit interacting closely with transmembrane helix 10 and the luminal loops of the P4 ATPase [19,20]. This association is required for both proper localization and activity of the pump but seems not to affect its substrate specificity [[21], [22], [23], [24], [25]]. Eukaryotes express several P4 ATPases that display different substrate specificities and subcellular localizations. Regarding the substrate specificity, P4 ATPases can be divided into three categories: enzymes that preferentially flip PS and to a lesser extent PE, enzymes that preferentially flip phosphatidylcholine (PC) and PE, and enzymes with a broad range of lipid substrates, including sphingolipids, lysophospholipids and synthetic alkylphospholipids [11,14].

While P4 ATPases only mediate inward-oriented transport of lipids, members of the ATP-binding cassette (ABC) superfamily of proteins operate as inward and outward directed lipid transporters [15,16,26]. Proteins in this superfamily share the same architecture, including two membrane-embedded transmembrane domains (TMD) required for substrate translocation across the membrane, and two cytoplasmic nucleotide-binding domains (NBD) that bind and hydrolyze ATP **(**Fig. 2B**)**. In eukaryotes, these four domains are organized either as full transporters or “half-transporters”, the latter class bearing single transmembrane and nucleotide-binding domain. The half-transporters need to form homo- or heterodimers to generate a functional ABC transporter [16,27]. Thus, ABC transporters feature a nucleotide-binding domain dimer that is stabilized by two ATP molecules, while P4-ATPases have a single location for ATP binding.

Sequence analysis of eukaryotic ABC transporters revealed that they can be divided into nine subfamilies (A–I) [28,29]. The TMD and NBD domains can display different topologies. In the forward topology, TMDs precede NBDs (TMD-NBD), whereas in the reverse arrangement TMDs follow NBDs (NBD-TMD) (Fig. 2B). This reverse topology is a characteristic feature of members of the ABCG subfamily in yeast. Notably, most members of the ABCC subfamily also have an extra N-terminal TMD composed of five transmembrane spans that precede the TMD-NBD domains. The eukaryotic members of different subfamilies are not exclusively located to the plasma membrane but also to peroxisomes, mitochondria and vacuoles. Similar to P4 ATPases, individual ABC transporters differ in their substrate specificities. Some utilize phospholipids among their substrates, others facilitate the transport of sterols [[30], [31], [32]]. Several of these ABC transporters have also been implicated in the development of drug resistance [30]. This observation implies that the mechanisms by which drugs are extrudedfrom cells are closely related to the mechanisms by which lipids are translocated across membranes.

A still unanswered question is how P4-ATPases and ABC transporters work to flip-flop lipids. In both the classical transport model for P4-ATPases and the “alternating access model” for ABC transporters, the lipid is proposed to gain access from one side of the membrane to a central cavity inside the transporter that is then closed and opened to the opposite side. This conformational switching of the membrane domain is driven by the binding of transport substrate and MgATP, followed by ATP hydrolysis [16]. The cavities within the transmembrane domains supposedly used to allocate the substrate during transport can be very different in size. In ABC transporters, the wide central cavity leaves plenty of space to accommodate a complete phospholipid molecule. Support for such a transport pathway has been provided by the structural characterization of the bacterial ABC transporter MsbA, an inner membrane transporter in Gram-negative *Escherichia coli* linked to the export of lipopolysaccharides. Structural analysis showed the lipopolysaccharide substrate entirely occluded inside MsbA [33]. Deviations from this substrate pathway must exist for ABC exporters transporting substrates which are too large to be accommodated in the cavity as it is proposed for PglK transporting lipid-linked oligosaccharides [34]. In this case, the cavity allows only access of phospholipid headgroup during transit through the membrane, while the hydrophobic hydrocarbon tails remain in contact with the hydrophobic core of the bilayer, in line with the “credit card model” [35]. Such a transport mechanism seems also likely for P4 ATPases which lack a big central cavity [19,20]. Further studies of different types of lipid flippases in complex with their lipid substrates are essential to establish whether several flipping mechanisms exist.

## 3 ATP-driven lipid transporters in fungi

A number of recent studies have led to the identification and characterization of ATP-dependent lipid transporters and their physiological functions in different fungal species, as summarized in Table 1, Table 2. In the following paragraphs, we will discuss different ATP-dependent lipid transporters and their potential physiological functions in model yeast species. We will then explore the functional aspects of lipid transporters in pathogenic fungi.

**Table 1:** Fungal P4 ATPases/Cdc50 complexes and their biological roles.

Species	α-subunit	β-subunit	Location	Substrate	Biological roles	Reference
***S. cerevisiae***	Drs2p	Cdc50p	TGN, EE, SV	PS, PE	SV biogenesis and segregation of cargo, TGN-endosomal trafficking, cell polarity, sterol homeostasis	[23,36,[43], [44], [45],^60^,[135], [136], [137]]
	Neo1p	–	Golgi, LE, PM^a^	n.i.	Vesicular transport, vacuole membrane fusion	[37,38,42,63,138]
	Dnf1p	Lem3p	PM, EE, TGN	PC, PE, (PS), LPC, LPE, LPS, GlcCer^b^, GalCer^b^	Endocytosis, cell polarity, protein sorting, endosomal trafficking	[37,39,46,47,[49], [50], [51],^139^,^140^]
	Dnf2p	Lem3p	PM, EE, TGN	PC, PE, (PS), LPC, LPE, GlcCer^b^, GalCer^b^	Endocytosis, protein sorting, endosomal trafficking	[39,46,47,[49], [50], [51],^139^,^140^]
	Dnf3p	Crf1p	TGN, SV	PC, PE	Vesicular transport	[37,43]
***C. neoformans***	Apt1p	Cdc50p^a^	Golgi^a^	n.i.	Antifungal resistance, vacuole organization, vesicle trafficking, iron acquisition, GXM secretion, lipid metabolism, intracellular survival, virulence in mice	[97,98,103,106,107]
	Apt2p	n.i	n.i	n.i	n.i	[106]
	Apt3p	n.i	n.i	n.i	Resistance to fluconazole and to brefeldin A	[106]
	Apt4p	n.i	n.i	n.i	n.i	[106]
***C. albicans***	Dnf1p	n.i.	n.i.	n.i.	Cooper resistance and tolerance to duramycin	[66]
	Drs2p	n.i.	n.i.	n.i.	Cooper resistance, tolerance to duramycin, fluconazole resistance and hyphal growth	[66,76]
	Neo1p	n.i.	n.i.	n.i.	Cooper resistance and tolerance to duramycin	[66]
***A. nidulans***	DnfAp	Cdc50p^a^	PM,Golgi, SPK (periphery)	PS	Vesicle trafficking, conidiation, pigmentation and hyphal growth	[90,91]
	DnfBp	Cdc50p^a^	PM, Golgi, SPK (core)	PS	Vesicle trafficking and sexual reproduction	[90,91]
	DnfDp	n.i.	Late Golgi	n.i.	Conidiation and conidiophore development	[92]
***M. grisea***	Pde1p	n.i.	n.i.	n.i.	Appressorium function	[93]
	MgAPT2p	n.i.	Golgi	n.i.	Exocytosis and plant tissue colonization	[94]

Abbreviations: **PM**: plasma membrane, **TGN**: trans-Golgi network, **SV**: secretory vesicles*,***EE**: Early endosome, **LE**: Late endosome, **SPK**: Spitzenkörper*,***GlcCer**: glucosylceramide, **GalCer**: galacotsylceramide, **PS**: phosphatidylserine, **PE:** phosphatidylethanolamine, **PC:** phosphatodylcholine, **LPE**: lysophosphatidylethanolamine, **LPC**: lysophosphatidylcholine, ^a^Putative. ^b^Glycolipids not endogenously produced by *S. cerevisiae,***n.i.**: not identified.

**Table 2:** Fungal ABC transporters involved in lipid transport and their biological roles.

Species	Protein	Location	Substrate	Biological role	Reference
***S. cerevisiae***	Pdr5p	Plasma membrane	PE	Externalization of lipids, drug efflux	[39,55,141]
	Yor1p	Plasma membrane	PE	Externalization of lipids	[39,55,59]
	Aus1p	Plasma membrane	Sterols	Import of exogenous sterols for anaerobic growth	[[56], [57], [58],^80^,^85^,^142^,^143^]
	Pdr11p	Plasma membrane	Sterols	Import of exogenous sterols for anaerobic growth	[[56], [57], [58],^85^,^143^,^144^]
	Ybt1p	Vacuole	PC	Transport of lipids and azoles into the vacuole	[59,74]
***C. albicans***	Cdr1p	Plasma membrane	PE, PC, PS	Externalization of lipids, drug efflux	[70,71,[145], [146], [147]]
	Cdr2p	Plasma membrane	PE, PC, PS	Externalization of lipids	[70,147]
	Cdr3p	Plasma membrane	PE, PC, PS	Internalization of lipids	[70]
	Mlt1p	Vacuole	PC	Transport of lipids and azoles into the vacuole, lipid homeostasis, endocytosis, secretory protease activity, tolerance to oxidative stress, hyphal development, virulence in mice	[[72], [73], [74]]
***C. glabrata***	Aus1p	Plasma membrane	Sterols	Import of exogenous sterols, mice kidney fungal burden, resistance to azoles in hypoxic conditions	[[78], [79], [80], [81],^148^]

Abbreviations: **PE**: phosphatidylethanolamine, **PC**: phosphatidylcholine, **PS**: phosphatidylserine.

### 3.1 *Saccharomyces cerevisiae* and *Schizosaccharomyces pombe*

The nonpathogenic yeast *S. cerevisiae* expresses five P4 ATPases, including Neo1p in the endosomal membranes, Drs2p and Dnf3p mostly in the trans-Golgi network, and Dnf1p and Dnf2p at the plasma membrane [[36], [37], [38], [39]]. While Neo1 has no known β subunit, Drs2p and Dnf3p interact with Cdc50p and Crf1p, respectively, and Dnf1p and Dnf2p both interact with Lem3p [23,40,41]. Neo1 is apparently implicated in the transport of PE and PS, but the lipid substrate for this P4 ATPase remains to be confirmed [42]. Dnf1p, Dnf2p, and Dnf3p have been identified as PE and PC flippases [39,43], while Drs2p transports PS and PE [[43], [44], [45]]. Dnf1p and Dnf2p in complex with their β subunit Lem3p were also found to transport alkylphospholipids, lysophosphatidylethanolamine, lysophosphatidylcholine, and monohexosyl glycosphingolipids [[46], [47], [48], [49], [50], [51]]. Notably, the Dnf2p ortholog of *S. pombe* transports glucosyl- and galatosylceramide (GlcCer and GalCer) but not PC and PE, suggesting that glycosphingolipid transport is a consolidated function of Dnf2p [49]. Considering that both *S. cerevisiae* and *S. pombe* are unable to synthesize GlcCer, it has been hypothesized that P4 ATPase-mediated transport of GlcCer in these organisms is related to sphingolipid scavenging from plant hosts. As GlcCer is known to be a virulence-associated molecule in many fungal pathogens [[52], [53], [54]], the elucidation of the transportation mechanisms of this lipid substrate is essential to understand its contribution to fungal pathogenesis.

Studies originally related to drug resistance identified two *S. cerevisiae* ABC transporters, the ABCC transporter Yor1p and the ABCG transporter Pdr5p [55]. In addition to amphiphilic drugs, these transporters mediate ATP-dependent movement of phospholipids from the inner to the outer leaflet of the plasma membrane [39,55]. Two other ABCG transporters, Aus1p and Pdr11p, operate as inward-directed transporters and facilitate the uptake of exogenous sterol, which is required for growth under anaerobic conditions [[56], [57], [58]]. The vacuolar ABCC transporter Ybt1p is required to import PC into vacuoles as part of choline recycling [59].

Several lines of evidence indicate that phospholipid translocation by ATP-driven transporters is required for membrane budding and endocytosis. Yeast cells lacking Dnf1p, Dnf2p and Drs2p display a cold-sensitive defect in endocytosis [39,60]. Loss of Drs2p results in a decrease in clathrin-coated vesicle budding from the *trans* Golgi network [45,60] and overexpression of ABC transporters with outward directed lipid translocase activity, resulting in defective endocytosis [55,61]. Members of both families appear to regulate the transbilayer lipid arrangement at the plasma membrane and other cellular locations, which is critical for budding of vesicles [62]. In agreement with this notion, the endosome-associated P4-ATPase Neo1p and the Golgi-localized P4-ATPase Dnf3 are required for protein trafficking between the Golgi complex, plasma membrane and endosomal / vacuolar system [37,38,63].

### 3.2 *Candida albicans* and *C. glabrata*

*Candida albicans* is a common human pathogenic fungus [64]. The number of individuals who are vulnerable to *Candida* infections has continuously increased as a consequence of the wide use of antibiotics, cancer therapy and solid organ transplantation, which highlights the need for a better comprehension on how *C. albicans* interacts with the host in their commensal and pathogenic stages [65].

Recent studies have shown that plasma membrane lipid asymmetry protects *C. albicans* from the toxic effects of copper [66], a metal used by the immune system to attack microbial pathogens [67]. Copper binds with high affinity to PS and PE promoting membrane damage and altered permeability [68,69]. Consequently, the exposure of these phospholipids at the cell surface upon deletion of P4 ATPase family members (*NEO1*, *DNF1*, *DRS2*) in *C. albicans* renders these cells sensitive to copper, with *drs2∆* cells exhibiting the strongest sensitivity. Conversely, cells lacking PS show resistance to copper, which indicates a major role for PS in copper sensitivity [66].

Some *C. albicans*ABCtransporters have also been shown to function as phospholipid translocators [70,71]. Interestingly, they differ in substrate specificity and the direction of phospholipid movement. While the ABCG transporters Cdr1p and Cdr2p are involved in the movement of PE,PC and PS from the inner to the outer leaflet of the plasma membrane and act in multidrug resistance, the ABCG transporter Cdr3p exhibits an inward-directed phospholipid translocase activity but does not participate in multidrug resistance [70]. The ABCC transporter Mlt1p of *C. albicans* has been shown to transport PC into the vacuolar lumen [72]. Deletion of the gene encoding this protein affects virulence-related traits, including hyphae formation, secretory protease activity and sensitivity to oxidative stress. This combination of affected virulence determinants culminated in attenuated virulence in mice [72,73]. Furthermore, both the Mlt1p transporter and its homologue in *S. cerevisiae* (Ybt1p) have been implicated in azole import into the vacuoles as an alternative to circumvent drug toxicity [74].

Resistance to the antifungal activity of fluconazole in *C. albicans* is a major issue, which has prompted the Centers for Disease Control and Prevention of the US to classify azole-resistant *Candida* species as a serious threat to human health [4,75]. Notably, *C. albicans* mutants lacking *DRS2* show hypersensitivity to fluconazole and altered hyphal growth [76]. In addition, the P4 ATPase subunit Cdc50p was reported as essential for antifungal drug resistance in *C. albicans.* Deletion of *CDC50* results in increased sensitivity to azoles, terbinafine and caspofungin, as well as to the membrane-perturbing agent sodium dodecyl sulfate. Furthermore, deletion of *CDC50* results in defective hyphal development and attenuated virulence in mouse model of systemic infection [77].

Recent studies revealed that sterol uptake can confer resistance to antifungal drugs, as inferred from the observation that mutant strains of *C. glabrata* lacking the ABCG transporter Aus1p exhibited reduced uptake of cholesterol and hypersensitivity to azoles [[78], [79], [80], [81]]. On the other hand, enhanced Aus1p expression and cholesterol uptake have been implicated in an azole-resistant phenotype [78,82,83]. *C. glabrata* can utilize exogenous cholesterol as a surrogate for ergosterol [84,85] when the ergosterol biosynthesis pathway is blocked, but also under regular conditions [79,86,87]. This promiscuous phenotype attenuates the effects of drugs that target ergosterol or ergosterol biosynthesis. In the same species, inhibition of ergosterol biosynthesis using fluconazole resulted in increased expression of the sterol influx transporter Aus1p. Cells lacking Aus1p did not show altered susceptibility to the non-azole antifungals amphotericin B, anidulafungin and caspofungin independently on the presence of exogenous sterols [81]. Thus, scavenging of exogenous sterols by sterol transporters may play an important role in antifungal resistance to azoles in pathogenic fungi.

### 3.3 Filamentous fungi

Invasive infections affecting mainly immunocompromised patients caused by filamentous fungi have increased over the last few decades, leading to fatal outcomes [88]. *Aspergillus fumigatus* is the primary cause of invasive aspergillosis in individuals with primary immunodeficiency, followed by *A. nidulans*, due to its ability to cause infection in patients with chronic granulomatous disease [89].

*A. nidulans* expresses four counterparts of the *S. cerevisiae* P4 ATPase family, including Dnf1/2p (DnfAp), Drs2p (DnfBp), Dnf3p, (DnfCp) and Neo1p (DnfDp) [90]. Little is known about the physiological functions of P4 ATPases in filamentous fungi. In *A. nidulans*, DnfAp and DnfBp localize to the Spitzenkörper [91]. This organelle, which is adjacent to the growing cell tip, is primarily composed of secretory vesicles that regulate fungal secretion and growth [90]. DnfAp is involved in asexual sporulation, pigmentation and polarized growth, while DnfBp potentially promotes sexual reproduction and has no role in conidiation; a double knockout of *DNFA* and *DNFB* is lethal in *A. nidulans*. [90]. Both proteins regulate PS asymmetry in *A. nidulans,* but localize to different regions of the Spitzenkörper. While DnfAp is concentrated in the peripheral region, DnfBp is distributed within the Spitzenkörper core, which indicates that these proteins may be present on different sets of vesicles [90,91]. Deletion of *DNFA* destabilizes the Spitzenkörper and cells depleted of the flippase β-subunit Cdc50 display defects in secretion, hyphal tip organization and morphology [91].

The relevance of DnfDp for *A. nidulans* growth and development has been recently demonstrated [92]. By analyzing mutants carrying single and pairwise deletions of P4 ATPases*,* Schultzhaus and collaborators found that deletion of *DNFD* (ortholog of the essential *S. cerevisiae NEO1* gene) resulted in a strong conidiation deficiency. Deletion of both *DNFB* and *DNFD* resulted in a lethal phenotype [92]. DnfDp localizes to the late Golgi and is also involved in the early stages of conidiophore development [92]. These results suggested that DnfDp is important in trafficking processes required for morphological changes during conidiation [92].

In *Magnaporthe grisea*, a plant pathogen, two P4 ATPases were found to be essential for virulence. A mutant strain lacking the *S. cerevisiae* Dnf3p ortholog *PDE1* is impaired in its ability to produce functional penetrating hyphae during plant infection. Moreover, *PDE1* is highly expressed during appressorium development, suggesting that Pde1p is essential for *M. grisea* virulence [93]. Likewise, the *S. cerevisiae* Drs2p ortholog *MgAPT2* is required for both foliar and root infection [94]. Mutants lacking *MgAPT2* are impaired in the secretion of numerous extracellular enzymes, display abnormal Golgi-like cisternae, and form abnormal penetrating hyphae [94]. These observations indicate that the regulation of membrane asymmetry by P4 ATPases is an important requirement for secretory processes and delivery of virulence-associated proteins in filamentous fungi.

## 4 ATP-driven lipid transporters in *C. neoformans*

The encapsulated basidiomycete *C. neoformans* is the major causative agent of meningoencephalitis in HIV-patients, leading to approximately 180,000 deaths annually, with 75% of the cases occurring in the Sub-Saharan Africa [95]. Cryptococcal meningitis is also a substantial problem for transplant recipients, patients with defects in cell-mediated immunity, and occasionally for immunocompetent individuals [96].

Sequence analysis of the *C. neoformans* genome identified four P4 ATPases, namely *APT1*, *APT2, APT3* and *APT4* [97]*.* Phylogenetic analysis revealed that Apt1p is closely related to Drs2p from *S. cerevisiae* and expression of *C. neoformans APT1* partially restored the growth of the *S. cerevisiae drs2∆* mutant strain [97]. In *C. neoformans,* the lack of Apt1p and its potential subunit, Cdc50p, affect many aspects of fungal physiology and virulence, as detailed in the next paragraphs.

Hu and Kronstad first demonstrated that deletion of *APT1* did not affect the well-known virulence factors of *C. neoformans*, including the polysaccharide capsule, melanin and urease [97]. However, *APT1* deletion impacted actin distribution, endocytosis, vesicle trafficking and fungal survival inside macrophages, possibly due to a higher sensitivity to oxidative and nitrosative stresses [97]. Although *APT1* deletion did not affect the release of extracellular vesicles, *apt1∆* cells produce extracellular vesicles with reduced concentration of glucuronoxylomannan (GXM) [98]. GXM, the major polysaccharide of the *C. neoformans* capsule [99], is synthesized in the Golgi and exported in vesicles that reach the extracellular space [100,101], together with other virulence-associated molecules [101,102].

The reduced amount of secreted GXM was accompanied by changes in the Golgi architecture and attenuated GXM synthesis in *apt1∆* cells [98,103]. Additionally, abnormalities in vacuolar membranes together with an accumulation of intra-vacuolar and pigment-containing vesicles were observed in *apt1∆* cells [103]. The *apt1∆* mutant secreted reduced amounts of GXM during macrophage infection and lung colonization in vivo [98]. Moreover, deletion of *APT1* resulted in virulence attenuation and inability to reach the brain in a mice model of infection [98].

**Fig. 3:**
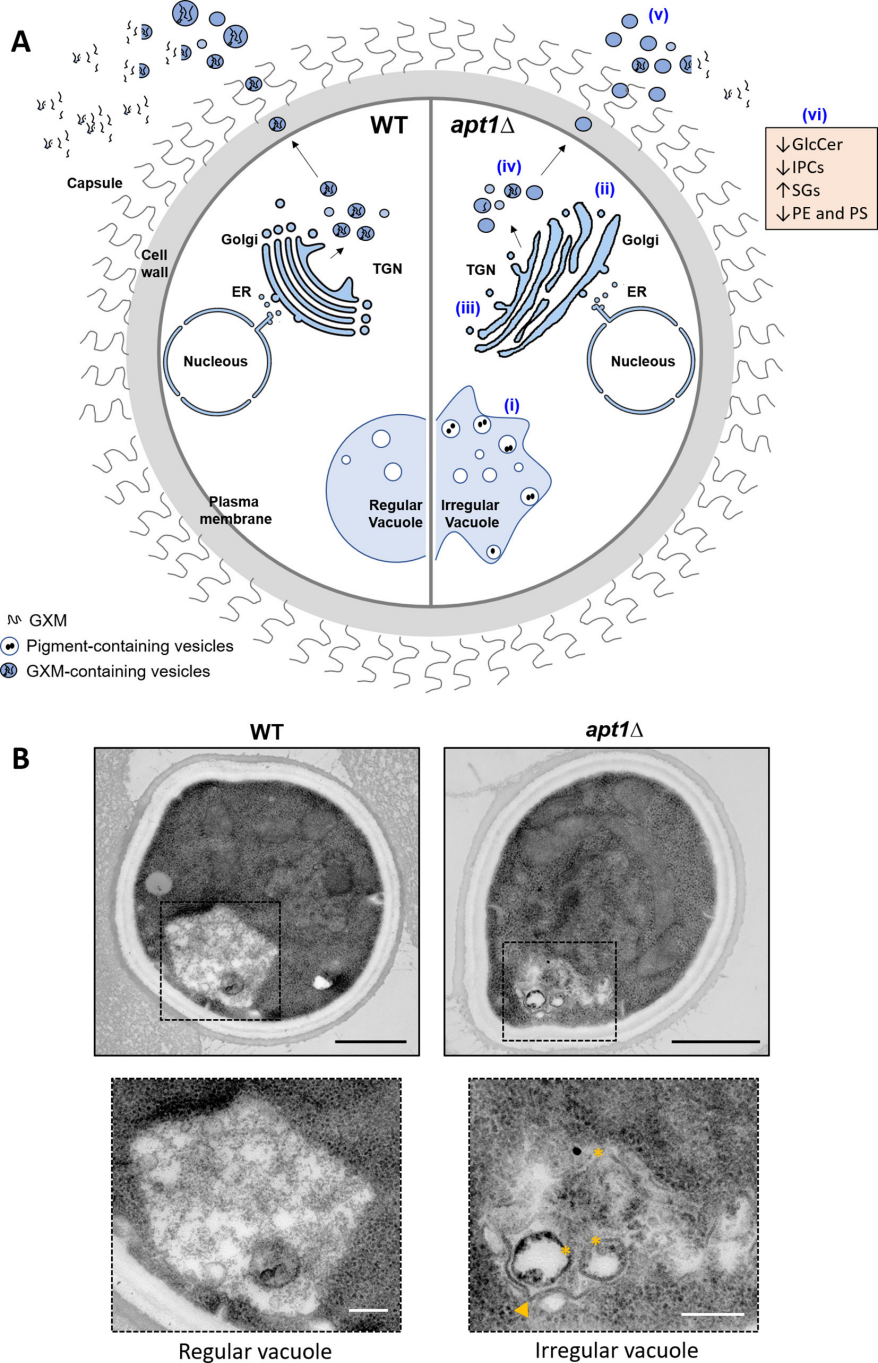
Illustration of the role of the P4 ATPase Apt1p in the *C. neoformans* physiology through the comparison of phenotypic traits of wild-type (WT) cells and a knockout strain (*apt1∆*). A) Apt1p is involved in regulating vacuolar morphology, distribution of pigment-containing vesicles (i) and Golgi architecture (ii). Lack of Apt1p results in higher sensibility to drugs targeting vesicle trafficking (monensin and brefeldin A), indicating altered ER-Golgi and trans-Golgi/post-Golgi complexes (iii). Deletion of *APT1* impacts GXM synthesis (iv) and its export to the extracellular environment (v). Apt1 is required for proper extracellular vesicles (EVs) dimensions and GXM concentration inside EVs (v). Deletion of *APT1* also affected lipid metabolism, with reduced levels of glucosylceramide (GlcCer), inositol phosphoryl ceramides (IPCs), phosphatidylserine (PS), phosphatidylethanolamine (PE), and accumulation of sterylglycosides (SGs) in total cell extracts (vi). B: Representative transmission electron microscopy images of *C. neoformans* WT and *apt1∆* cells. Boxed areas illustrating vacuolar morphology were magnified. Mutant cells showed abnormal vacuoles, suggesting defects in membrane dynamics (arrowhead) and accumulation of pigment-containing vesicles (asterisks). Scales bar represent 1 μm and 250 nm (magnified fields).

Further investigation provided evidence that deletion of *APT1* affected the synthesis of virulence-associated lipids. The absence of *C. neoformans* Apt1p resulted in altered lipid metabolism, with reduced levels of GlcCer and inositol phosphoryl ceramides in association to accumulation of sterylglycosides [103]. The absence of GlcCer or even changes in GlcCer structure led to loss of virulence and impaired dissemination to the brain in *C. neoformans* [52,54]. Similarly, downregulation of inositol phosphoryl ceramide synthase 1 *(IPC1)* affected fungal growth inside macrophages and resulted in impaired pathogenesis and reduced fungal burden in the cerebral spinal fluid of infected rabbits [104]. The accumulation of sterylglycosides was also reported to be relevant for immunological control of animal cryptococcosis [105]. Substrate specificity and subcellular localization of Apt1p, as well as its relationship with phospholipid synthases, remain to be explored*.* The overall effects of *APT1* deletion on *C. neoformans* are illustrated in Fig. 3.

Two independent studies demonstrated a key role for the P4 ATPase subunit Cdc50p as an important regulator of *C. neoformans* virulence. *C. neoformans* Cdc50p shares properties with both *S. cerevisiae* Cdc50p and Lem3p [106]. The protein is located to the ER, plasma membrane and endosome-like structures [106,107]. Cells lacking Cdc50p expose PS on the outer leaflet of the plasma membrane and are highly sensitive to trafficking inhibitors, as well as to the echinocandin caspofungin [107]. *C. neoformans* is intrinsically resistant to echinocandins and the results provided by Huang and collaborators suggest that the *CDC50* gene may be associated to cryptococcal resistance to this antifungal agent, since plasma membrane defects due to *CDC50* deletion led to enhanced caspofungin penetration into the cell [107].

Brown and colleagues demonstrated that Cdc50p influences the activation of the Rim signaling pathway, suggesting that Cdc50p-depending cellular processes contribute to the timing and intensity of Rim 101 cleavage and localization [108]. The rim 101 pathway is involved in the mechanisms by which *C. neoformans* recognizes and responds to changes in the extracellular pH, as well as in its evasion of the host immune response [109]. These results are likely linked to the well reported growth defects of *cdc50Δ* and *apt1Δ* strains at alkaline pH [106,107] and suggest that plasma membrane asymmetry is involved in the response to altered extracellular pH.

*C. neoformans* mutant cells lacking *CDC50* shared phenotypical features with strains that had the *APT1*, *APT2, APT3* or *APT4* genes individually deleted, including cell wall integrity, with unaffected sensitivity to agents that challenge the cell wall, such as congo red, calcofluor white and caffeine. Notably, *cdc50∆* and *apt1∆* cells share several phenotypic traits, including attenuated survival in macrophages, reduced GXM secretion, growth defect in alkaline pH, and increased sensitivity to the iron-chelating drug curcumin, to trafficking inhibitors (brefeldin A and monensin) and to antifungal drugs, including azoles, amphotericin B and cinnamycin [97,98,106,107]. The latter is an antifungal peptide that targets PE exposed on the outer leaflet of the plasma membrane. Additionally, in a murine model of infection, both *cdc50∆* and *apt1∆* strains were hypovirulent and unable to reach the brain, which is the fatal outcome of cryptococcosis [97,98,106,107]. These data imply that Cdc50p serves as a β subunit for Apt1p to form a functional heterodimeric flippase complex which may represent a novel target for antifungal development. Whether Cdc50p is the only β subunit and if it can also interact with APT2, 3 and 4 remains to be explored.

Little is known about the biological roles of the *APT1* paralog genes *APT2, APT3, and APT4*.The comparison of phenotype characteristics between mutants lacking *APT1–4* and the *CDC50* expression showed that Apt2p, Apt3p, and Apt4p do not play a role in iron acquisition, resistance to curcumin and growth at alkaline pH, contrasting to what was observed for Apt1p and Cdc50p [106]. Intriguingly, the *apt1∆* and *apt3∆* mutants showed increased sensitivity to the trafficking inhibitor brefeldin A, although to a lesser extent when compared to the *cdc50∆* mutant*.* Additionally, Apt1p and Apt3p appeared to make redundant physiological contributions because the double mutant (*apt1∆apt3∆*) was more sensitive to brefeldin A than each single mutant. It was also observed that *apt1∆* and *apt3∆* cells display increased sensitivity to fluconazole, with the *cdc50∆* mutant showing the most evident phenotypic alterations [106]. In this context, the relevance of the *APT2–4* in *C. neoformans* physiology and virulence remains to be investigated. The major roles of *CDC50* and *APT1–4* in *C. neoformans* virulence-associated features are summarized in Table3.

**Table 3:** *C. neoformans* virulence−associated features in *cdc50∆ and apt1–4∆* cells.

Features	*cdc50∆*	*apt1∆*	*apt2∆*	*apt3∆*	*apt4∆*	References
Melanin	−/0	0	0	0	0	[97,106,107]
Capsule	+/0	0	0	0	0	[97,98,106,107]
Growth at 37 °C	−/0	0	0	0	0	[97,106,107]
Growth in salt stress	−	0	0	0	0	[97,106,107]
Growth in alkaline pH (9.0)	−	−	0	0	0	[[106], [107], [108]]
Growth in acidic pH (4.0)	−	0	0	0	0	[106]
GXM Secretion	−	−	nt	nt	nt	[98,107]
Lipid Metabolism	nt	+/−	nt	nt	nt	[103]
Sensitivity to nitrosative and oxidative stresses	0	+	nt	nt	nt	[97,106,107]
Release of extracellular vesicles	nt	0	nt	nt	nt	[98]
Virulence in murine model	−	−	nt	nt	nt	[97,98,106,107]
Intracellular proliferation in macrophages	−	−	nt	nt	nt	[97,106,107]
Membrane integrity	−	0	0	0	0	[97,106,107]
Cell Wall Integrity	0	0	0	0	0	[97,106,107]
Iron acquisition	−	−	0	0	0	[106]
PS Exposure (Annexin V binding)	+	0	nt	nt	nt	[98,107]
Sensitivity to Cinnamycin (PE asymmetry)	+	+	nt	nt	nt	[97,106]
Sensitivity to Miltefosine	+	nt	nt	nt	nt	[106]
Sensitivity to Brefeldin A (Trafficking inhibitor) inhibitor)	+	+	0	+	0	[97,106]
Sensitivity to Monensin (Trafficking inhibitor)	+	+	nt	nt	nt	[97,106]
Sensitivity to Curcumin (Iron chelator)	+	+	0	0	0	[106]
Sensitivity to Fluconazole	+	+	0	+	0	[97,106,107]
Sensitivity to Amphotericin B	+	+	nt	nt	nt	[97,107]
Sensitivity do Caspofungin	+	0	0	0	0	[106,107]

Abbreviations: (**+**) Enhanced; (**−**) Reduced; (**0**) Not affected; (**nt**) Not tested; (**+/−**) Enhanced or reduced depending on lipid class; (**+/0**) Enhanced or not affected, **(−/0)** Reduced or not affect (contrast in different reports); **GXM**: Glucuronoxylomannan; **PE**: phosphatidylethanolamine; **PS**: phosphatidylserine.

## 5 ATP- independent lipid transporters in fungi

Recent studies in *A. fumigatus* identified an ATP-independent lipid transporter of the TMEM16 family [110,111]. Most members of the TMEM16 protein family are Ca^2+^-regulated lipid scramblases [15,112,113] that facilitate the bidirectional movement of phospholipids across membranes. However, the role of TMEM16 in *A. fumigatus* physiology and pathogenicity is still unknown. Another report identified single nucleotide polymorphisms (SNPs) in a scramblase family of isolates from a patient suffering from persistent and recurrent invasive aspergillosis [114]. This report suggests that SNPs in *A. fumigatus* scramblases, together with other proteins, could have arisen during the course of host infection. The involvement of scramblases in this microevolution process still needs to be explored.

ATP-independent lipid scramblases have also been associated with the physiopathogenesis of *Cryptococcus* spp. Mutant cells of *C. gattii*, in which a gene encoding a putative scramblase (*AIM25*) was disrupted, exhibited alterations in extracellular vesicle formation, GXM secretion, and surface architecture [115,116].

First insight into the transport mechanism of this family of scramblases was provided by resolving the crystal structure of TMEM16 from the filamentous fungi *Nectria haematococca* [117]. The structure is that of a dimer, the native state in which TMEM16 proteins are isolated from cells. Each monomer has a remarkable large groove in its transmembrane domain that would allow accommodating the headgroup of a transiting phospholipid, suggesting that scrambling occurs via the ‟credit-card” mechanism. Recent cryo-electron microscopy structures of TMEM16 from *A. fumigatus* (afTMEM16) showed that the opening of the lipid pathway in response to Ca^2+^-binding also leads to a visible thinning of the membrane that could facilitate scrambling by destabilizing the local membrane order [111]. Indeed, recent findings suggest that scrambling mediated by TMEM16 proteins occurs through dual mechanisms: lipids can traverse the membrane either by passing through the hydrophilic grooves, or outside of the groove due to the presence of local defects in the packing of the membrane [118].

## 6 Membrane lipid organization as a target for antifungal drugs

The prevalence of fungal infections and the acquisition of drug resistance are increasing over the years, indicating the need for new strategies for identifying targets for antifungals development [4]. Multidrug resistance has been documented in both laboratory and clinical settings, and resistant outbreaks have been reported in hospitals [4,[119], [120], [121]]. The newest class of antifungals of clinical use is the echinocandins, which dates of 2002. However, as the use of echinocandins is becoming widespread, isolates with reduced susceptibility have been reported [122]. In the case of *C. neoformans,* the intrinsic resistance to echinocandins limits treatment options to compounds that target membrane components, including ergosterol and the enzymes required for its biosynthesis [4]. The recent discovery that the P4 ATPase subunit Cdc50p is associated to the resistance phenotype to caspofungin in *C. neoformans* indicates that lipid transporters and their interacting proteins are promising targets for antifungals [8,107].

Additional membrane proteins related to the control of phospholipid asymmetry are involved in the effectivity of antifungals. Among those, Rta3p represent a member of the Rta1p-like lipid-translocating exporter family. *RTA3* is coordinately upregulated with *CDR1* and *CDR2* in azole-resistant clinical isolates of *C. albicans* [123], and overexpression of Rta3p in azole-susceptible strains resulted in increased tolerance to this antifungal [124]. Rta3p localizes to the plasma membrane, and regulates biofilm formation and PC asymmetry across the plasma membrane [124,125]. However, its precise molecular functions remain to be established.

Altering the lipid composition in fungal membranes by limiting phospholipid synthesis has been recently addressed in *C. albicans* and *C. neoformans*. In *C. albicans*, the PS synthase Cho1p was required for fungal viability and virulence [126,127]. *CHO1* mutation affected mitochondrial function, filamentous growth, and perturbed cell wall integrity and thickness [126]. Defects in the cell wall of the *cho1∆/∆* mutant were associated to an overexposure of β-1,3- glucan in yeast and hyphal forms. The *cho1∆/∆* mutant also manifested higher binding to the dectin-1 receptor and elicited TNF-α secretion by macrophages [128]. In *C. neoformans*, the Cho1p is located to the ER and regulates mitochondrial function, possibly by contributing to the maintenance of mitochondrial membrane integrity. PS was essential for *C. neoformans* viability, suggesting that phospholipid synthases are fundamental for the physiology of this pathogen [129]. These studies highlight the relevance of fungal enzymes involved in phospholipid synthesis and distribution for fungal physiology and pathogenicity and indicates potential targets for antifungal development based on the absence of mammalian homologs to some of these enzymes [130].

One key issue for future efforts is to identify how to modulate and/or inhibit specifically fungal lipid transporters during the different stages of fungal pathogenesis. Understanding the mechanisms by which lipid transporters recognize and translocate substrates, the interaction partners and regulation, and how their activity modulates the expression of numerous virulence factors in fungi will be of great help to the development of therapeutic strategies to control fungal infections. The possibility of changing the structure of fungal membranes through perturbation of phospholipid asymmetry, e.g. by synthetic scramblases [[131], [132], [133], [134]], or to design inhibitors for lipid flippases or phospholipid synthases could represent novel approaches for the development of membrane-based antifungals. Novel molecules, alone or in combination with classical antifungals, could potentially overcome resistance and enhance the efficacy of antifungal treatment.

## Funding

JR acknowledges support from the Brazilian agency Conselho Nacional de Desenvolvimento Científico e Tecnológico(CNPq, grant: 381575/2018–7) and the Research Explorer Ruhr program funded by Germany's Excellence Initiative [DFG GSC 98/3]. VKAS is supported by Coordenação de Aperfeiçoamento de Pessoal de Nível Superior(CAPES, finance code 001). MLR is supported by grants from CNPq (grants 405520/2018-2, 440015/2018-9 and 301304/2017-3) and Fiocruz (grants VPPCB-007-FIO-18-2-57 and VPPIS-001-FIO-18-66). LN is supported by grants from CNPq (grant 311179/2017-7 and 408711/2016-7). The authors also acknowledge support from the Instituto Nacional de Ciência e Tecnologia de Inovação em Doenças de Populações Negligenciadas(INCT-IDPN). MLR is currently on leave from the position of Associate Professor at the Microbiology Institute of the Federal University of Rio de Janeiro, Brazil.

## Declaration of Competing Interest

The authors declare no conflict of interest.
